# Rumors of Its Disassembly Have Been Greatly Exaggerated: The Secret Life of the Synaptonemal Complex at the Centromeres

**DOI:** 10.1371/journal.pgen.1002807

**Published:** 2012-06-28

**Authors:** Satomi Takeo, R. Scott Hawley

**Affiliations:** 1 Stowers Institute for Medical Research, Kansas City, Missouri, United States of America; 2 Graduate School of Life and Environmental Sciences, University of Tsukuba, Ibaraki, Japan; 3 Department of Molecular and Integrative Physiology, Kansas University Medical Center, Kansas City, Kansas, United States of America; The University of North Carolina at Chapel Hill, United States of America

The synaptonemal complex (SC) and the centromeres have long been thought to play their roles at quite different, non-overlapping periods during meiosis. The SC is viewed as a hallmark structure that connects homologous chromosomes and is functionally critical for early prophase (zygotene and pachytene), while centromeres are known to assemble the kinetochore, act as a target for microtubules, and direct chromosome movement during anaphase I and II.

**Figure 1:**
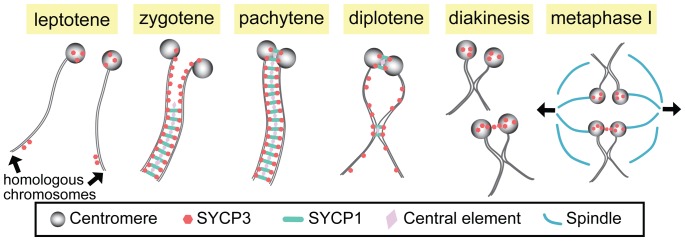
Synapsis and centromere pairing in mouse spermatocytes. Homologous chromosomes are not paired in leptotene, but SYCP3 starts to localize to centromeres and arms. In zygotene, synapsis initiates from the non-centromeric regions and progresses as SCYP1 and central element proteins zip up SYCP3-containing chromosome axes. At late zygotene, centromeres are at last paired and synapsed, and full SC is formed along the entire chromosome in pachytene. In diplotene, the SC disassembles except at the sites where centromere pairing persists and chiasmata are formed. At diakinesis/metaphase I, SYCP3 proteins are accumulated on the centromere and a small fraction of homologous centromeres are linked by SYCP3-containing stretches, which may function in bi-orientation of homologous centromeres and attachment to the spindles for the first meiotic division.

However, several recent studies have suggested much earlier functions for centromeres (at least in some organisms) and detailed post-pachytene functions of the SC. First, studies in budding yeast, *Drosophila*, and higher plants provide evidence that centromeres play an important role in the initiation of synapsis in these systems [1]–[5]. Second, building on earlier work [6], [7], recent studies have clearly demonstrated that in both yeast and flies the SC persists at the centromeres long after the end of pachytene—at least until late prophase [1], [8], [9]—and that these regions of centromeric pairing and synapsis play important roles in mediating segregation at anaphase I (see below). In this issue of *PLoS Genetics*, two studies of mammalian spermatocytes [10], [11] demonstrate that while the centromeres are not involved in synaptic initiation in mammals, stretches of the SC at paired centromeres and at sites of crossing over do indeed persist beyond the breakdown of synapsis along the chromosome arms at the end of pachytene (see Figure 1).

The SC is a proteinaceous railroad-track–like structure that assembles in early meiotic prophase immediately following or concomitant with homolog pairing. This assembly process is often referred to as homolog synapsis. Although the SC is structurally complex, it may be thought of as consisting primarily of three components: lateral elements that run along the entire length of each homolog (and in mammals contain the SYCP3 protein); transverse filaments that, like the teeth of a zipper, serve to connect the two lateral elements of the SC (and include the mammalian SYCP1 protein); and a set of proteins located at the center of the SC (cleverly referred to as central element proteins).

The question thus arises as to the function of these short regions of SC that survive the end of pachytene. Studies in yeast have clearly shown that the maintenance of centromeric pairing by short regions of the SC plays a role in mediating the segregation of both exchange and non-exchange chromosome segregation at meiosis I [8], [9]. Similar data demonstrate that persistent pericentromeric pairings also play a critical role in mediating the segregation of non-exchange homologs in *Drosophila* [12], [13].

In this issue of *PLoS Genetics*, the Pezza and Hunter groups show that the SC at mammalian centromeres also persists beyond the breakdown of synapsis along the chromosome arms at the end of pachytene in mouse spermatocytes [10], [11]. Indeed, the centromeres are the last sites at which SC is observed. Both groups also demonstrate that the persistent pairing of centromeric regions is dependent on the transverse filament protein SYCP1. Moreover, Qiao et al. [11] characterize the structure of the SC at centromeres by structured illumination microscopy (SIM), revealing that the SC stretches at the centromere represent bona fide tripartite SC in which transverse filaments connect two lateral elements.

Unlike SC breakdown along the chromosome arms following the end of pachytene, the much later dissolution of the SC at the centromeres may be a temporally complex process in which at least one SC component remains at the centromeres. Consistent with previous observations, both Bisig et al. [10] and Qiao et al. [11] show that while SYCP1 becomes undetectable prior to Nuclear Envelope Breakdown, SYCP3 remains associated with the centromeres until at least anaphase I in males. Qiao et al. also suggest that in some cases the persistence of SYCP3 at the centromere may provide a “bridge between the two homologous kinetochores” at diakinesis/metaphase I. Studies in other organisms support the view that such SYCP3 bridges may play important roles in facilitating segregation of achiasmate sex chromosomes [14].

However, centromeres do not appear to function as synaptic initiation sites in mammals. Indeed, both Bisig et al. and Qiao et al. demonstrate that centromeres are the last to pair, doing so only at zygotene–pachytene transition. Qiao et al. also use Rnf212 (Zip3 homolog) knock-out mice to explore whether or not centromeres can be “forced” to become synaptic initiation sites. In yeast *zip3* mutants, synapsis occurs predominantly at the centromeres [2]. In *Rnf212^−/−^* spermatocytes, however, synapsis does not initiate at centromeres even though SC-associated centromere pairing at diplotene in the mutants was similar to that observed in wild-type. These data show clearly that, unlike the situations in yeast, flies, and higher plants, centromeres in the mouse do not have the capacity to initiate synapsis—indeed, they are extremely delayed in terms of their ability to initiate synapsis.

Finally, Qiao et al. demonstrate that remnants of SC also persist beyond pachytene at the sites of chiasma formation where they may regulate the necessary local remodeling of the homolog axes. In the absence of SYCP1, chiasma-like structures are still observed at diplotene (despite the inability of this genotype to generate normal crossovers), but these aberrant structures are more numerous and often exhibit fused axial elements as well as fusions of telomeric axes. The authors interpret these data to mean that prior to chiasma formation, the SYCP3-based axes are stabilized by the presence of the central region of the SC.

The take-home messages from these stories are that the SC's functions don't end with the disappearance of the majority of the structure at the end pachytene, nor are they limited to synapsis and recombination. Such findings are a complement to the demonstration that in at least some organisms (although not in mammals) the centromeres act as sites of the synaptic initiation. Moreover, SC structure at the centromeres may be somewhat different from that of the so-called normal euchromatic SC, and at least some SC components (such as SYCP3) may have functions that persist even after dissolution of the complete SC. Thus the SC needs to be considered as a rather flexible and adaptable structure that functions far more widely (both temporally and mechanistically) than previously thought. Given their roles in synaptic initiation in some organisms, the same may be true of centromeres. It's a fascinating partnership.
